# Mechanisms of Neuroprotection by Quercetin: Counteracting Oxidative Stress and More

**DOI:** 10.1155/2016/2986796

**Published:** 2016-01-24

**Authors:** Lucio G. Costa, Jacqueline M. Garrick, Pamela J. Roquè, Claudia Pellacani

**Affiliations:** ^1^Department of Environmental and Occupational Health Sciences, University of Washington, Seattle, WA 98105, USA; ^2^Department of Neuroscience, University of Parma Medical School, 43100 Parma, Italy

## Abstract

Increasing interest has recently focused on determining whether several natural compounds, collectively referred to as nutraceuticals, may exert neuroprotective actions in the developing, adult, and aging nervous system. Quercetin, a polyphenol widely present in nature, has received the most attention in this regard. Several studies* in vitro*, in experimental animals and in humans, have provided supportive evidence for neuroprotective effects of quercetin, either against neurotoxic chemicals or in various models of neuronal injury and neurodegenerative diseases. The exact mechanisms of such protective effects remain elusive, though many hypotheses have been formulated. In addition to a possible direct antioxidant effect, quercetin may also act by stimulating cellular defenses against oxidative stress. Two such pathways include the induction of Nrf2-ARE and induction of the antioxidant/anti-inflammatory enzyme paraoxonase 2 (PON2). In addition, quercetin has been shown to activate sirtuins (SIRT1), to induce autophagy, and to act as a phytoestrogen, all mechanisms by which quercetin may provide its neuroprotection.

## 1. Introduction

Quercetin (3,3′,4′,5,7-pentahydroxyflavone) is a common flavonol found in many fruits and vegetables such as apples, berries, onions, and capers [1]. Together with flavones, anthocyanidins, and various other compounds, flavonols belong to the class of flavonoids, which in turn represent a major class of polyphenols [2]. The dietary intake of all flavonoids has been estimated at about 200–350 mg/day, while intake of flavonols is about 20 mg/day, of which quercetin accounts for nearly 50%, with a daily intake of approximately 10 mg/day [3]. A recent study carried out in Japan supported these estimates, as daily intake of quercetin was determined to be 16 mg [4]. As quercetin is present in fruits and vegetables, high consumption of such foods can increase intake to over 200 mg/day. Among vegetables, the highest levels of quercetin have been found in onions (*Allium cepa* L.), asparagus (*Asparagus officinalis* L.), and red leaf lettuce (*Lactuca sativa* L.), with lower levels in broccoli, green peppers, peas, and tomatoes. Apples are the fruits with the highest quercetin content, together with cherries and various berries (Table 1). The quercetin in foods is not present as aglycone (i.e., without sugar groups), but as quercetin glycosides [3]. Quercetin aglycone is also sold as a dietary supplement, with a recommended dosage of 1 g/day [5].

## 2. Quercetin: Absorption, Metabolism, and Bioavailability

**Figure 1:**
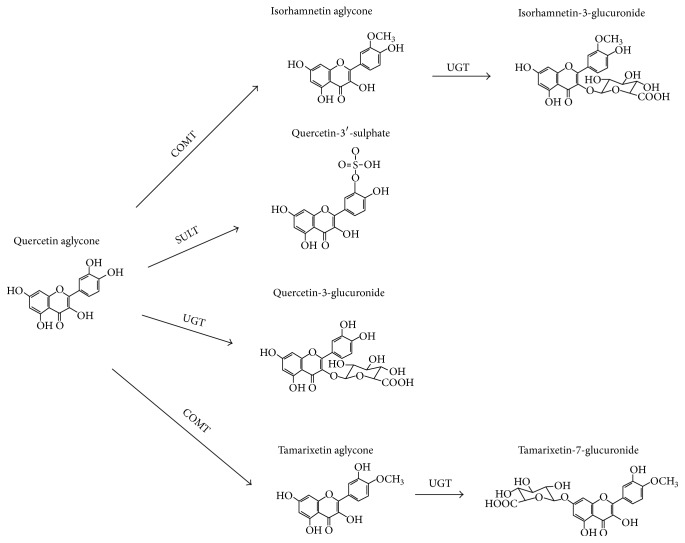
Structure of quercetin and of some of its principal metabolites (see text for further details).

Quercetin glycosides present in foods (e.g., onions), such as quercetin glucoside, quercetin galactoside, or quercetin arabinoside, are deglycosylated to quercetin aglycone prior to passive absorption in the small intestine [6]. Enzymes involved in this first reaction are lactase phlorizin hydrolase (a beta-glucosidase) and/or gut microbiota-derived beta-glucosidase, depending on the nature of the glycoside [2, 6]. The quercetin aglycone undergoes significant and extensive biotransformation reactions to form glucuronidated, sulfated, and methylated metabolites, indicating an involvement of the phase II enzymes UGT (uridine 5′-diphospho-glucuronosyltransferase), SULT (sulfotransferase), and COMT (catechol-O-methyltransferase).  Figure 1 illustrates examples of quercetin metabolites [3′-O-methyl-quercetin (isorhamnetin), quercetin-3-O-glucuronide, 3′-O-methylquercetin-O-glucuronide, and quercetin-3′-O-sulfate] derived from these biotransformation reactions.

Studies in rats and pigs have shown that quercetin distributes to several tissues, particularly lung, kidney, colon, and liver, with lower levels in brain [7]. Total quercetin derived from the diet is normally present in plasma in the nanomolar range (<100 nM) but can be increased to the low micromolar range after supplementation of quercetin aglycone or glycosides [8, 9]. The half-life of quercetin ranges between 11 and 28 h, suggesting the possibility of significantly increasing plasma concentration upon supplementation [8, 10]. In general, bioavailability of quercetin is low, and it varies significantly among individuals, though the underlying mechanisms are poorly understood [6].

Only limited amounts of quercetin aglycone are found after ingestion of quercetin, though there is some controversy on this issue (see, e.g., [11, 12]), and methylated, sulfated, and glucuronide metabolites are the most prominent moieties found in plasma. Studies have shown that glucuronidated metabolites have antioxidant abilities* in vitro* and* in vivo* [13, 14]. Additional biological effects of methylated and sulfate metabolites have been reported [15–17], though some studies have failed to observe an effect of quercetin metabolites [18]. Of interest is also the observation that conjugated quercetin can enter the cell (erythrocyte), where it is converted to its nonconjugated form [19].

An important issue for the potential use of quercetin* in vivo* is whether it passes the blood-brain barrier (BBB) and what concentrations of quercetin and/or its metabolites are present in brain tissue.* In vitro* studies with BBB models consistently indicate that quercetin enters the brain [20–22]. Upon administration of quercetin* in vivo* to rats and pigs, low levels (from picomolar to nanomolar) are found in brain tissue [7, 21, 23]. Of interest in this regard are the recent successful efforts to increase bioavailability of quercetin [24]. In particular, the formulation of quercetin in lipid nanoparticles significantly increases its penetration into the brain [25, 26]. Additionally, coadministration of quercetin and alpha-tocopherol has been shown to increase the transport of quercetin across the blood-brain barrier [27].

Quercetin has an unremarkable toxicological profile, as evidenced by animal and human studies [5, 24]. Similar to other polyphenols, reported beneficial effects of quercetin include effects on cardiovascular diseases, cancer, infections, inflammatory processes, gastrointestinal tract function, diabetes (reviewed in [12, 24, 28]), and nervous system disorders, which are discussed below. Previous relevant reviews on the potential for quercetin to exert neuroprotection have been published [29, 30].

## 3. Neuroprotective Effects of Quercetin:* In Vitro* Studies

*In vitro* studies in neuronal cell lines and in primary neurons have shown that quercetin, at low micromolar concentrations, antagonizes cell toxicity induced by various oxidants (e.g., hydrogen peroxide, linoleic acid hydroperoxide) and other neurotoxic molecules believed to act by inducing oxidative stress (e.g., 6-hydroxydopamine and N-methyl-4-phenyl-1,2,3,6-tetrahydropyridinium) [29, 31–34]. A recent study showed that quercetin glycosides (rutin, isoquercitrin) were capable of antagonizing changes in gene expression induced by 6-hydroxydopamine in PC12 cells [35]. In isolated rat brain mitochondria, the toxicity of the anticancer drug oxaliplatin was antagonized by quercetin, which significantly reduced oxidative stress [36]. Protection of neuronal cells from the toxicity of amyloid beta peptide toxicity has also been reported [37].

Experimental conditions (e.g., end-points, duration of incubation) vary significantly in published* in vitro* studies; however, quercetin exerts neuroprotection* in vitro* at concentrations that are in the micromolar range [29], which is higher than the concentration found upon* in vivo* administration. In addition, most of the absorbed quercetin is present as metabolites, which have undergone only limited testing* in vitro*. Nevertheless, a number of glucuronidated, methylated, and sulfated quercetin metabolites have been shown to have neuroprotective actions* in vitro* [14–17], though negative results have also been reported [18].

## 4. Neuroprotective Effects of Quercetin: Human and Animal Studies* In Vivo*

Extensive evidence supports the notion that diets rich in polyphenols and/or supplementation with specific compounds provide beneficial health effects. In particular, polyphenols have been shown to exert protective actions in several pathological conditions such as cardiovascular disease, metabolic disorders, obesity, diabetes, infections, cancer, and neurotoxic/neurodegenerative processes [12, 44–46].

Specific evidence exists on the neuroprotective effects of quercetin [29]. Several studies show that quercetin can exert neuroprotection and antagonize oxidative stress when administered* in vivo*. For example, oral quercetin (0.5–50 mg/kg) was shown to protect rodents from oxidative stress and neurotoxicity induced by various neurotoxic insults [21, 25]. Among metals, quercetin has been shown to provide protection against the neurotoxicity of lead, methylmercury, and tungsten [38, 40, 43]. The neurotoxicity of polychlorinated biphenyls, of the insecticide endosulfan, and of MPTP (1-methyl-4-phenyl-1,2,3,6-tetrahydropyridine) has also been shown to be reduced by quercetin* in vivo* [39, 41, 42].  Table 2 shows some details on the effects of quercetin treatments toward neurotoxicity induced by these compounds. Quercetin also antagonized cognitive impairment induced by feeding mice a high fat diet [47]. It was also neuroprotective in models of intracerebral hemorrhage in rats [48] and protected the retina from apoptotic damage due to ischemia-reperfusion injury in a rat model [49]. Of relevance are also some recent findings showing that quercetin ameliorates Alzheimer's disease pathology and related cognitive deficits in an aged triple transgenic Alzheimer's disease mouse model [50]. Of additional interest are the findings that a combined oral supplementation of quercetin and fish oil enhanced neuroprotection in rats exposed to 3-nitropropionic acid or chronically treated with the insecticide rotenone [51, 52]. The latter is considered an animal model of Parkinson's disease.

## 5. Counteracting Oxidative Stress as a General Mechanism of Neuroprotection by Quercetin

Oxidative stress is recognized as an important factor in a variety of neurodegenerative diseases, as a mediator of the adverse effects of a number of neurotoxicants, and as a mechanism for age-related degenerative processes [53–55]. Oxidative stress occurs when reactive oxygen species (ROS) accumulate in cells, from either excessive production or insufficient neutralization, causing damage to proteins, lipids, and DNA. Mitochondria are a major contributor of cellular ROS; ROS produced in the mitochondria can also target the electron transport chain (e.g., complex I), resulting in a cycle where ROS production increases, followed by ATP depletion and ultimately cell death [56]. Based on these premises, the identification of novel compounds which can counteract oxidative stress as potential therapeutics is a very active area of research [57]. Natural compounds have received much attention in this regard, and polyphenols such as quercetin have been the most investigated [29, 58].

### 5.1. Quercetin as a Direct Antioxidant

Quercetin is a potent scavenger of ROS, such as O_2_^∙−^, and of RNS (reactive nitrogen species), such as NO and ONOO [28]. The antioxidant capacity of quercetin has been ascribed to the presence of two pharmacophores within the molecule that have the optimal configuration for free radical scavenging, that is, the catechol group in the B ring and the OH group at position 3 [28]. At concentrations of 5 to 50 *μ*M quercetin can directly scavenge ROS* in vitro* [59]. However, the concentration of quercetin expected to be present in the brain would likely be in the nanomolar range, well below that necessary to exert an appreciable direct antioxidant effect. In contrast, other important antioxidants, such as glutathione and vitamin C, are present at millimolar concentrations [22]. Thus, despite its potent antioxidant capacity* in vitro*, it is unlikely that neuroprotective effects of quercetin observed* in vivo* are due to a direct antioxidant action. Rather, it has been suggested that quercetin and/or its metabolites may act by modulating the cell's own antioxidant defense mechanisms [60, 61], suggesting that quercetin may act as a prooxidant, rather than an antioxidant [62, 63]. A mild degree of oxidative stress may indeed increase the cell's own antioxidant defenses, resulting in overall cytoprotection.

### 5.2. Modulation of Antioxidant Pathways by Quercetin

As indicated, quercetin may have prooxidant, rather than antioxidant, properties [28, 62, 63]. In the process of antioxidant activities, quercetin oxidizes into various oxidation products, including semiquinone radicals and quinones [28]. Interestingly, such compounds may mediate the toxic effects of quercetin observed in certain conditions [28, 63]. Evidence is emerging to support hormetic roles for small increases in membrane and mitochondrial oxidative stress [64]. Hormesis, which is generally defined as a dose-response phenomenon characterized by low-dose stimulation and high-dose inhibition, also includes the phenomenon of “preconditioning,” in which exposure to a low dose of an agent that is toxic at high doses induces an adaptive, potentially beneficial effect on the cell or organism if exposed to a subsequent and more massive dose of the same or related stressor agent [65].

#### 5.2.1. The Nrf2-ARE Pathway

Nrf2 [nuclear factor (erythroid-derived 2)-like 2] is an important regulator of cellular defense against oxidative stress. Under physiological conditions, Nrf2 is sequestered in the cytoplasm by the protein Keap1 (Kelch-like ECH-associated protein 1) with Cullin 3-base E3 ligase, resulting in the ubiquitination of the Nrf2 protein and its targeting for proteasomal degradation [66–68]. Keap1 has several cysteine residues which make it act as a molecular switch, responding to electrophiles and ROS with a conformational change which releases Nrf2 [66]. Dissociated Nrf2 translocates into the nucleus where it binds to small Maf proteins. The formed heterodimer binds to cis-acting antioxidant response elements (ARE) and thereby promotes the transcription of a broad range of phase II and antioxidant genes [67, 68]. Proteins under control of the Nrf2-ARE pathway include heme oxygenase-1, glutamate cysteine ligase, glutathione S-transferases, glutathione peroxidase, superoxide dismutase, catalase, sulfiredoxin, and thioredoxin [66, 68].

Activation of the Nrf2-ARE pathway provides neuroprotection against oxidative damage and cell death. More recent evidence also suggests that the Nrf2-ARE pathway may modulate the formation and degradation of misfolded protein aggregates which are present in various neurodegenerative diseases (Parkinson's, Alzheimer's, and Huntington's diseases and amyotrophic lateral sclerosis) [68]. For example, studies with tert-butylhydroquinone, a prototype Nrf2 inducer, have shown that activation of the Nrf2-ARE pathway confers protection against neurotoxicity induced by amyloid beta and 3-nitropropionic acid [66–69].

Quercetin has been shown to counteract oxidative stress-induced cellular damage by activating the Nrf2-ARE pathway [33, 59, 70], and similar effects have been reported for dihydroquercetin [67]. Additionally, other nutraceuticals (e.g., kaempferol, pterostilbene) have been shown to interact synergistically with quercetin in this regard [59]. Pathways involved in the activation of Nrf2 include the ERK and JNK signaling [67], and they in turn are activated by stress stimuli including mild oxidative stress, suggesting that quercetin may act as a neurohormetic phytochemical [71].

#### 5.2.2. The Paraoxonase 2 (PON2) Pathway

PON2 is a member of the paraoxonase family of genes which also includes PON1 and PON3. PON2 does not have esterase activity but is a potent lactonase [72] and plays a significant role in atherosclerosis [73]. In contrast to PON1 and PON3, which are present primarily in the liver and in blood, PON2 is a ubiquitously expressed intracellular enzyme [74–76]. PON2 mRNA has been found in mouse and human brain, and PON2 protein has been detected in mouse [75–77], rat, human, and monkey brain ([78]; Costa et al., unpublished). In mouse brain the highest levels of PON2 protein were found in dopaminergic regions, while, at the cellular level, PON2 is higher in astrocytes than in neurons or microglia. Subcellular distribution studies have shown that PON2 is localized primarily in the mitochondria [76, 79]. Interestingly, female mice express higher levels of PON2 than males, and this sex difference has been also seen in other species (rat, human, and monkey) ([76, 78, 80]; Costa et al., unpublished). This may be related to a positive modulatory effect by estrogens. Indeed, 17-beta-estradiol increases PON2 expression, possibly by activating the alpha estrogen receptor [78].

PON2 has been shown to exert an antioxidant effect, which is believed to play a major role in preventing the atherosclerotic process and in neuroprotection [76, 79–81]. The preponderant localization of PON2 in mitochondria would support a role for this enzyme in protecting cells from oxidative damage. In HeLa cells, PON2 has been shown to bind to coenzyme Q10 that associates with complex III in mitochondria, and PON2 deficiency causes mitochondrial dysfunction [79]. In human endothelial cells PON2 has been shown to reduce, indirectly but specifically, the release of superoxide from the inner mitochondrial membrane, without affecting levels of other radicals such as hydrogen peroxide and peroxynitrite [82]. Of relevance is also the fact that mitochondria, together with the cytoplasm and the nucleus, are preferential accumulation sites for quercetin in cells [83].

The cytotoxicity of two known oxidants, hydrogen peroxide (H_2_O_2_) and 2,3-dimethoxy-1,4-naphthoquinone (DMNQ), is much higher in cells from PON2^−/−^ mice [76]. Similarly, striatal astrocytes and neurons from male mice (which express low PON2 levels) are more sensitive to H_2_O_2_- and DMNQ-induced oxidative stress and ensuing cytotoxicity than female cells [78].

Given the antioxidant actions of PON2 in the CNS, a positive modulation of PON2 may result in neuroprotection [80]. In macrophages, PON2 expression is increased by oxidative stress [84], and in vascular cells an endoplasmic reticulum stress element-like sequence was found to be present in the promoter region of PON2 [81]. Various compounds have been shown to upregulate PON2 expression in various tissues or cell types (see details in [85]).

Quercetin was reported to increase PON2 mRNA and protein in macrophages* in vitro*, though administration of 150 mg/day to human volunteers for six weeks was without effect [86]. Another recent study examined the induction of PON2 by quercetin in brain cells* in vitro* [34]. Quercetin increases PON2 protein expression in mouse striatal astrocytes and neurons and in macrophages. The effect of quercetin is antagonized by SP600125, an inhibitor of the JNK/AP-1 pathway, but not by the PPAR gamma inhibitor GW9662. One possibility is that quercetin may induce a very low level of oxidative stress [62, 87], which in turn would modulate the JNK/AP-1 pathway [88], causing an increase in PON2 expression. Alternatively, given the effects of estradiol on PON2 expression [78], quercetin may induce PON2 expression by virtue of its phytoestrogen activity ([17, 89]; see also Section 9).

Independent of the underlying mechanism(s), the ability of quercetin to induce PON2 may play a role in its neuroprotective actions. Indeed, in striatal astrocytes from wild-type mice, quercetin abolished the increase in ROS levels and the cytotoxicity induced by H_2_O_2_ or DMNQ, providing a 4-fold protection (Table 3). In contrast, in cells from PON2^−/−^ mice the toxicity of H_2_O_2_ and DMNQ was decreased by less than 2-fold; this partial protection may be due to Nrf2-ARE induction [34].

## 6. Induction of Autophagy

An additional mechanism for quercetin neuroprotection relates to the modulation of autophagy. Autophagy (from the Greek “to eat oneself”) refers to the cellular degradative pathways that involve delivery of the cytoplasmatic cargo to the lysosomes [90–92]. Autophagy (macroautophagy) is a multistep process involving the formation of double membrane structures, the autophagosomes, which then fuse with lysosomes. The content of the resulting autophagolysosomes (misfolded proteins, cellular metabolic waste) is then degraded by hydrolytic enzymes. Autophagy is also important for removal of damaged mitochondria and of normal mitochondria undergoing turnover, in a process known as mitophagy. The integrity of the CNS is highly dependent on normal basal autophagy, as damaged organelles and misfolded proteins would accumulate in neurons unless they are successfully removed [90]. Rapamycin, an inhibitor of mTOR (mammalian target of rapamycin) activity, is a potent inducer of autophagy and acts as a neuroprotector [92, 93]. In contrast, deletion of key autophagy genes (*Atg5*,* Atg7*) causes severe neurodegeneration [94]. Stimulation of autophagy in the CNS would thus lead to neuroprotection, as has been shown for various compounds [92]. Quercetin has been shown to alleviate cell damage caused in Schwann cells by high glucose by inducing autophagy [95]. Similarly, in* C. elegans*, the neurotoxicity of amyloid beta 1-42 is antagonized by quercetin through induction of autophagy [96].

## 7. Modulation of Sirtuins

An additional field of interest with regard to the mechanisms of neuroprotection provided by quercetin is that of sirtuins. These proteins (in mammals there are seven, named SIRT1 to SIRT7) are involved in a variety of cellular and molecular processes and pathways, with distinct cellular localization and molecular targets [97]. Of these, SIRT1 predominantly localizes in the nucleus and acts as a deacetylase for histones and other targets. SIRT1 protects cells from apoptosis and promotes differentiation of stem cells. SIRT2 is prevalently in the cytoplasm and has been found to accumulate in neurons, while other SIRTs localize primarily in the mitochondria [97]. The neuroprotective effects of quercetin may also involve activation of SIRT1, which would lead to suppression of Bax-dependent apoptosis and repression of multiple proapoptotic transcription factors. A recent example of the effects of quercetin on this pathway is represented by findings showing that quercetin inhibits* herpes simplex* virus type 1-induced neurodegeneration by activating SIRT1 [98].

## 8. Modulation of Neuroinflammation

Neuroinflammation is emerging as playing a most relevant role in neurodevelopmental and neurodegenerative disorders and thus represents a potential important target for therapeutic interventions [99, 100]. Compounds that may antagonize microglia activation and reduce the release of proinflammatory cytokines would be of much relevance. Some isoflavones are suggested to reduce microglial activation and subsequent release of proinflammatory factors [101], and polyphenols may have beneficial anti-inflammatory properties [45]. Quercetin has been shown to reduce lipopolysaccharide- (LPS-) induced nitric oxide release from a mouse neuroglia cell line [102]; a similar effect on LPS-induced proinflammatory cytokines was reported in another mouse microglial cell line [103]. In addition, quercetin also inhibits cytokine production by astrocytes [104]. The cellular/molecular mechanisms for the anti-inflammatory effects of quercetin are not known, but a possible pathway may be related to induction of PON2 which has anti-inflammatory activity in addition to its antioxidant activity.

## 9. Quercetin as a Phytoestrogen

As indicated earlier, induction of PON2 expression by quercetin may be ascribed to its phytoestrogen activity. The classification of quercetin as a phytoestrogen is still controversial [105, 106], and so is the potential involvement of estrogen alpha or beta receptors in its action [89, 107]. Nevertheless, both the quercetin aglycone and its glucuronide have been recently shown to possess estrogenic activity and to activate estrogen receptor alpha [17]. The neuroprotective actions of estrogens are well known, though the exact mechanisms are not fully understood [108, 109]. Interestingly, the protective effect of estradiol was absent in cells from PON2^−/−^ mice, suggesting that a major mechanism of estrogen neuroprotection may be represented by induction of PON2 [78]. Identical findings have been reported with regard to quercetin [34]. Thus, additional studies on the role of estrogen receptor pathways in neuroprotection by quercetin would be relevant.

## 10. Conclusion

There is an increasing interest for the potential neuroprotective effects of quercetin and other nutraceuticals. This brief review has focused on mechanisms related to the ability of quercetin to counteract oxidative stress-mediated neurotoxicity and on some additional potential mechanisms of neuroprotection. However, further targets for biological activity are to be expected, for example, related to signal transduction pathways, proteasome function, mitochondrial integrity, and so on [24]. An important issue to consider as part of a discussion on the beneficial effects of quercetin remains to be that of increasing its access to the CNS, and notable progress has been made in recent years in this regard. In addition, the potential role played by quercetin metabolites should be examined more systematically, as only limited information is available [110].

## Figures and Tables

**Table 1:** Quercetin content in selected vegetables and fruits (mg/100 g).

Onion	11–33
Lettuce (red)	10–30
Pepper	10–30
Broccoli	3–5
Tomato	2–4
Asparagus	7–20
Peas	14

Apple	2–5
Cherry	1–3
Blueberry	5

Adapted from [1, 4, 6].

**Table 2:** Neuroprotection by quercetin against neurotoxicants *in vivo*.

Animal	Neurotoxicant	Quercetin	Effect of quercetin	Ref.
M, F Wistar rats	Lead (0.2% in water through pre- and postnatal development)	30 mg/kg/d for 7 d starting at PND 60	Decreased lipid perox. in hippocampus; partial reversal of LTP	[38]
M Wistar rats	PCBs (Aroclor 1254) 2 mg/kg/d for 30 d i.p.	50 mg/kg/d for 30 d, orally	Decreased ox. stress in cerebellum; reduced dopaminergic toxicity	[39]
M Wistar rats	MeHg 30 mg/kg/d for 45 d, orally	0.5, 5, and 50 mg/kg/d for 45 d, orally	Decreased reduction of GSH, GPx (5, 50 mg/kg)	[40]
M C57BL/6 mice	MPTP 30 mg/kg/d for 4 d (10–14 of Q)	50, 100, and 200 mg/kg/d for 14 d	Diminished reduction of DA levels, SOD, and GPx	[41]
M Wistar rats	Endosulfan 2 mg/kg/d, for 6 d, orally	10 mg/kg/d, for 6 d, orally	Diminished lipid perox. and mitochondria swelling	[42]
M Wistar rats	Tungsten 100 ppm in water for 3 mo.	0.3 mM/d for 3 mo., orally	Reduced oxidative stress	[43]

F: female; GPx: glutathione peroxidase; GSH: glutathione; M: male; MeHg: methylmercury; MPTP: 1-methyl-4-phenyl-1,2,3,6-tetrahydropyridine; PCBs: polychlorinated biphenyls; PND: postnatal day; SOD: superoxide dismutase.

**Table 3:** Protective effect of quercetin against oxidative stress and cytotoxicity in mouse striatal astrocytes.

	ROS (% of basal)	Cytotoxicity (IC_50_, *μ*M)
Control		
H_2_O_2_	630	39
DMNQ	695	37

+ Quercetin		
H_2_O_2_	130^*∗*^	157^*∗*^
DMNQ	115^*∗*^	131^*∗*^

For ROS measurements, control or quercetin-pretreated cells (24 h, 20 *μ*M) were exposed to either oxidant for 30 min. For cytotoxicity (assessed by the MTT assay) control or quercetin pretreated cells were exposed for 24 h to 4-5 concentrations of oxidants. ^*∗*^Significantly different from control, *p* < 0.01. Adapted from [34].
